# Double-Edged Sword: Interleukin-2 Promotes T Regulatory Cell Differentiation but Also Expands Interleukin-13- and Interferon-γ-Producing CD8^+^ T Cells *via* STAT6-GATA-3 Axis in Systemic Lupus Erythematosus

**DOI:** 10.3389/fimmu.2021.635531

**Published:** 2021-03-02

**Authors:** Hiroshi Kato, Andras Perl

**Affiliations:** Division of Rheumatology, Departments of Medicine, Microbiology and Immunology, and Biochemistry and Molecular Biology, College of Medicine, State University of New York, Upstate Medical University, Syracuse, NY, United States

**Keywords:** systemic lupus erythematosus, interleukin-2 (IL-2), interleukin-13 (IL-13), interferon-γ (IFN-γ), T regulatory (Treg) cell, CD8^+^ T cells, STAT6, GATA-3

## Abstract

Interleukin-2 (IL-2) expands the depleted T regulatory (Treg) cell population, and it has emerged as a potential therapy in systemic lupus erythematosus (SLE). However, IL-2 administration may involve the risk of expanding unwanted pro-inflammatory cells. We herein studied the effects of IL-2 on pro-inflammatory cytokine production by CD4^+^ and CD8^+^ T cells in parallel with Treg development following CD3/CD28 co-stimulation. While Treg cells are depleted in SLE patients, their CD4^+^ T cells were poised to receive and activate IL-2 signaling as evidenced by upregulation of CD25 and enhanced IL-2-incued STAT5 phosphorylation during Treg differentiation. In patients with SLE, however, IL-2 also expanded CD8^+^ T cells capable of producing interleukin-5, interkeukin-13 (IL-13), and interferon-γ (IFN-γ) that occurred with enhanced expression of GATA-3 and phosphorylation of STAT6 but not STAT5. Our data pinpoint a safety signal for systemic administration of IL-2 and challenges a long-held conceptual platform of type 1 and 2 cytokine antagonism by newly documenting the IL-2-dependent development of IL-13 and IFN-γ double-positive (IL-13^+^IFNγ^+^) CD8^+^ T cells in SLE.

## Introduction

Systemic lupus erythematosus (SLE) is an autoimmune disease characterized by aberrant T- and B-cell activation culminating in a production of antinuclear antibodies (1). Among numerous immune dysregulation pathways implicated in the pathogenesis, the depletion of T regulatory (Treg) cells has emerged as an important mediator of the disease (2–4). In this respect, interleukin (IL)-2 elicits T regulatory (Treg) cell differentiation in association with phosphorylation of STAT5 and its binding to the *Foxp3* gene (5). These findings along with IL-2 deficiency in lupus patients (6) yielded the notion that supplementation of IL-2 could restore the immune tolerance by expanding the Treg cell population. Indeed, low dose IL-2 therapy was shown to expand Treg cells and ameliorate the lupus disease activity (7).

Non-selective administration of IL-2, however, poses a concern for potential expansion of unwanted pro-inflammatory cells in view of its pleiotropic functions, in particular, its role as a T-cell growth factor (8). In fact, IL-2 induces Th1 and Th2 differentiation in a STAT5-dependent manner (9, 10). In addition, IL-2 elicits the differentiation of naïve CD8^+^ T cells into effector and memory cytotoxic T cells along with the induction of interferon (IFN)-γ, perforin, and granzymes (11).

While extensive evidence underpins the essentiality of CD4^+^ and CD4^-^CD8^-^ double-negative T cells in lupus pathogenesis (12–14), roles for CD8^+^ T cells have also been increasingly recognized. CD8^+^ T cells during a lupus flare exhibit more prominent cytotoxic phenotype and functions than during remission, and the frequency of such cells correlates with the SLE disease activity index (SLEDAI) score (15). Tubulointerstitial nephritis associated with CD8^+^ T cell infiltrates confers an increased risk for progressive lupus nephritis (16). With regard to the type of immune response mediated by T cells, it remains controversial whether SLE is driven by type 1 or type 2 immunity given the various animal models showing discrepant findings. In humans, some studies showed increased IL-4, but decreased IFN-γ in lupus patients (17), whereas others indicate the importance of IFN-γ in diffuse proliferative lupus nephritis (18). SLE patients with higher SLEDAI score have lower IFN-γ but higher IL-4 expression than those with lower SLEDAI score (19). Another type 2 cytokine, IL-13, shares many biological functions with IL-4, as exemplified by when human IL-13 elicits B-cell proliferation and its immunoglobulin production (20–22). In addition to the contribution of IL-13 to asthma and allergic disorders (23, 24), it is important to note that GATA-3-dependent IL-13 production by CD8^+^ T cells promotes fibrosis in systemic sclerosis (25, 26). While our understanding of lupus T cell biology has been rapidly evolving, it is unknown how IL-2 affects the lineage-specification of lupus T cells, and whether IL-13 plays a role in immune dysregulation in SLE.

In this study, we evaluated the effects of IL-2 on pro-inflammatory cytokine expression in SLE CD4^+^ and CD8^+^ T cells in comparison to those on Treg cell development. While Treg population is depleted in SLE patients, their CD4^+^ T cells were primed to receive and activate IL-2 signaling as evidenced by upregulation of CD25 and enhanced IL-2-induced STAT5 phosphorylation during Treg differentiation. On the other hand, SLE CD8^+^ T cells produced greater amount of IL-13 and IFN-γ than CD4^+^ T cells in an IL-2-dependent manner. In addition, IL-2 expanded the IL-13-producing lupus CD8^+^ T cells that also expressed IL-5 and IFN-γ in association with STAT6 phosphorylation and GATA-3 expression, but not with STAT5 phosphorylation. Our data conveys a clear safety signal in further pursuit of systemic administration of IL-2, and supports the rationale for Treg-cell-targeted delivery of IL-2 in the treatment of SLE.

## Materials and Method

### Human Subjects

In total, 33 patients with SLE fulfilling the American College of Rheumatology diagnostic criteria (27) were studied. In each experiment, peripheral blood was obtained from SLE patients (all female) and healthy control (HC) subjects who were matched to the patients by age (within 10 years), sex, and ethnic background. Age of study participants was 44.8 ± 2.0 (mean ± SD) years in SLE, and 44.2 ± 1.9 years in HC subjects. Disease activity was assessed by the SLEDAI scores (28), which ranged from 0 to 34 (mean ± SD: 6.24 ± 1.17). Mean daily prednisone dose was 6.52 ± 1.62 mg. Immunosuppressive drugs taken by the study subjects included hydroxychloroquine (N=31), methotrexate (N=1), mycophenolate mofetil (N=10), mycophenolic acid (N=1), azathioprine (N=5), cyclosporine (N=1), tacrolimus (N=1), belimumab (N=6), and abatacept (N=1). The study was approved by the Institutional Review Board at the SUNY Upstate Medical University.

### Isolation of Untouched T Cells and Cell Culture

Peripheral blood mononuclear cells (PBMCs) were isolated by using Ficoll Histopaque gradient (GE Health Care Bio-Sciences). CD3^+^ T cells were isolated by negative selection using untouched human T cell isolation kit (Life technologies, Cat# 11344D). Purity of CD3^+^ T cells was confirmed to be above 97%. Cells were cultured in RPMI culture media with 10% FCS, 1% Penicillin/Streptomycin, and 1% L-glutamine (all from Corning CellGro except for FCS, which was from Gibco) for 3 days, and stained with PE Cy7-conjugated anti-CD4 (Clone: SK3, Cat# 557852, RRID : AB_396897) and phycoerythrine (PE)-conjugated anti-CD25 (Clone: M-A251, Cat# 555432, RRID : AB_395826, both from BD Biosciences). The cells were permeabilized as per the manufacturer’s instructions and stained with AF-647-conjugated anti-FoxP3 (Biolegend, Clone: 150D, Cat# 320014, RRID : AB_439750).

### Isolation of CD4^+^ and CD8^+^ T Cells and Cell Culture

CD4^+^ and CD8^+^ T cells were isolated by negative selection using human CD4^+^ T cell enrichment (Cat# 15062) and CD8^+^ T cell isolation kits (Cat# 17953, both from STEMCELL), respectively. Cells were cultured for 3 days in the presence of plate-bound anti-CD3 (anti TCR ϵ hybridoma from ATCC) and soluble anti-CD28 (1 μg/ml, BD Biosciences, Clone: CD28.2, Cat# 555725, RRID : AB_396068) in the presence or absence of IL-2 (100 IU/ml, Peprotech, Cat# 200-02) or anti-IL-2 (100 or 1000 ng/ml, R&D Systems, Cat# MAB202, RRID : AB_2264789).

### Intracellular Staining

Cells were pre-incubated with PMA (5 ng/ml) and ionomycin (500 ng/ml) for 6 h, and with brefeldin A for 5 h (10 μg/ml; all from Sigma-Aldrich). For cytokine detection, FITC-conjugated anti–IFN-γ (Clone: B27, Cat# 554700, RRID : AB_395517), PE-conjugated anti-IL-5 (Clone: JES1-39D10, Cat# 559332, RRID : AB_397229), or IL-17 (Clone: SCPL1362, Cat# 560436, RRID : AB_1645514), BV711 conjugated anti-IL-13 (Clone: JES10-5A2, Cat# 564288, RRID : AB_2738731), and allophycocyanin-conjugated anti-IL-4 (Clone: 8D4-8, Cat# 560671, RRID : AB_1727546) or IL-21 (Clone: 3A3-N2.1, Cat# 562043, RRID : AB_10896655, all from BD Biosciences) were used alone or together. Isotype control Abs included FITC-conjugated mouse IgG1 κ (Clone: MOPC-21, Cat# 551954, RRID : AB_394297), PE-conjugated rat IgG2a κ (Clone: R35-95, Cat# 559317, RRID : AB_10050484), BV711-conjugated rat IgG1 κ (Clone: R3-34, Cat# 563283, RRID : AB_2869482), and Alexa Fluor (AF)-647-conjugated mouse IgG1 κ (Clone: MOPC-21, Cat# 557732, RRID : AB_396840, all from BD Biosciences).

### T Regulatory Cell Polarization

Naive CD4^+^ T cells were isolated from SLE and matched HC subjects by using Human Naive CD4^+^ T cell Enrichment Kit (STEMCELL, Cat# 19555). The purity of naive CD4^+^ T cells as defined by the proportion of CD4^+^CD45RA^+^CD62L^+^ cells was above 99%. Cells were cultured for 72 h in the presence of anti-CD3/CD28 and TGF-β (5 ng/ml, Peprotech, Cat# 100-21) with IL-2 (50 IU/ml) or anti-IL-2 (100 or 1000 ng/ml). Cells were stained with FITC-conjugated anti-CD25 (Clone: M-A251, Cat# 555431, RRID : AB_395825) and AF-647-conjugated anti-FoxP3 (Clone: 259D/C7, Cat# 560045, RRID : AB_1645411 both from BD Biosciences). Frequency of CD4^+^CD25^+^FOXP3^+^ cells was determined by flow cytometry. In other experiments, CD4^+^ T cells isolated from matched SLE and HC subjects were cultured for 3 days in the presence of anti-CD3/CD28 and TGF-β (20 ng/ml) with or without IL-2 (100 IU/ml) or anti-IL-2 (100 ng/ml).

### Immunoblotting

Using lysates of CD4^+^ T cells cultured under Treg-polarizing conditions, total STAT5 (Clone: A-9, Cat# sc-74442, RRID : AB_1129711) and its phosphorylation at tyrosine 694 (pSTAT5^Y694^, Clone: C11C5, Cat# 9359, RRID : AB_823649) were detected by immunoblotting. Using lysates of CD8^+^ T cells cultured in the presence or absence of IL-2 or anti-IL-2, phosphorylation of STAT5 at tyrosine 694, phosphorylation of STAT6 at tyrosine 641 (pSTAT6^Y641^, Clone: C11A12, Cat# 9364, RRID : AB_2271227), and expression of GATA-3 (Clone: HG3-31, Cat# sc-268, RRID : AB_2108591) were determined by immunoblotting (anti-STAT5 and anti-GATA-3 were from Santa Cruz Biotechnology whereas the remainder was from Cell Signaling Technology). The signal intensity was normalized to that of actin (Millipore, Clone: C4, Cat# MAB1501, RRID : AB_2223041).

### Statistical Analysis

Student t test was performed for comparison of phenotype between two groups with two-tailed p values < 0.05 considered significant. Two-way ANOVA was followed by Bonferroni’s posttest for multiple comparisons using Prism 8 software (GraphPad, La Jolla, CA). Association of two variables was determined by Pearson’s and Spearman’s correlation analyses.

## Results

### Systemic Lupus Erythematosus CD4+ T Cells Are Poised to Receive Interleukin-2 Signaling During T Regulatory Cell Differentiation

**Figure 1:**
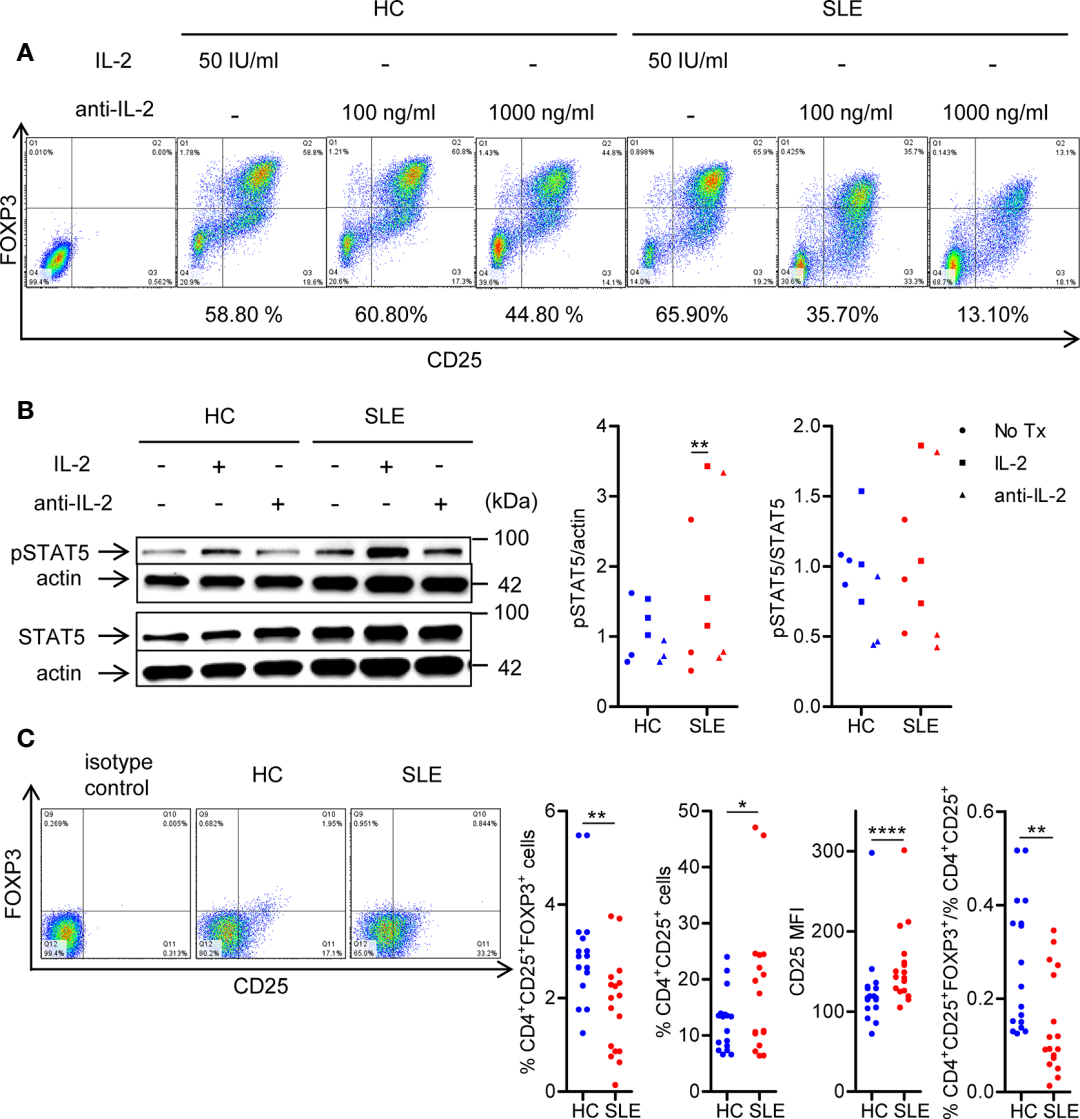
SLE CD4^+^ T cells are poised to activate IL-2 signaling during Treg differentiation. **(A)** Naïve CD4^+^ T cells were isolated from a systemic lupus erythematosus (SLE) patient and matched healthy control (HC) subject, and cultured for 3 days in the presence of anti-CD3/CD28 and TGF-β (5 ng/ml) with IL-2 (50 IU/ml) or anti-IL-2 (100 or 1,000 ng/ml). The frequency of CD4^+^CD25^+^FOXP3^+^ cells was determined by flow cytometry. Numbers below the plots represent the frequency of CD4^+^CD25^+^FOXP3^+^ Tregs. The dot plots on the left end represent isotype control staining. **(B)** CD4^+^ T cells isolated from matched SLE and HC subjects were cultured for 3 days in the presence of anti-CD3/CD28 and TGF-β (20 ng/ml) with or without IL-2 (100 IU/ml) or anti-IL-2 (100 ng/ml). Total STAT5 and its phosphorylation at tyrosine 694 were detected by immunoblotting. Representative immunoblot staining (left panel). The signal intensity of phospho-STAT5 and total STAT5 was normalized to that of actin. The normalized pSTAT5 signal intensity (middle panel) and the ratio of normalized pSTAT5 signal intensity over normalized STAT5 signal intensity (right panel) from 3 pairs of matched HC and SLE subjects. **(C)** Untouched T cells from matched SLE and HC subjects were cultured for 3 days without anti-CD3/CD28 stimulation. Expression of CD25 and FOXP3 in CD4^+^ cells were determined by flow cytometry. Representative flow cytometry dot plots are shown (left panel). Cumulative data of frequency of CD4^+^CD25^+^FOXP3^+^ and CD4^+^CD25^+^ cells, mean fluorescence intensity (MFI) of CD25 expression in CD4^+^ T cells, and the proportion of CD4^+^CD25^+^FOXP3^+^ cells among CD4^+^CD25^+^ cells from 17 pairs of matched SLE and HC subjects (right panel). Data were analyzed by a paired two-tailed t-test (*p<0.05, **p<0.01, ****p<0.0001).

To confirm the essentiality of IL-2 signaling in Treg differentiation in SLE, naïve CD4^+^ T cells were cultured under Treg-polarizing conditions in the presence or absence of IL-2 or anti-IL-2. IL-2 blockade abrogated Treg differentiation both in SLE and healthy control subjects (**Figure 1A**). Of note, supplementation of IL-2 induced STAT5 phosphorylation in SLE CD4^+^ T cells, but not in healthy control CD4^+^ cells (**Figure 1B**). Such a heightened sensitivity to IL-2 prompted us to examine the expression of IL-2 receptor α chain (CD25) on SLE CD4^+^ T cells (29). Instead of immunophenotyping cells immediately after isolation, untouched CD3^+^ T cells were rested in culture media without anti-CD3/CD28 stimulation for 3 days to eliminate the effects of immunosuppressive drugs that the study subjects had received. Although CD4^+^CD25^+^FOXP3^+^ Treg population was depleted, CD25 was upregulated on lupus CD4^+^ T cells, resulting in diminished proportion of CD4^+^CD25^+^FOXP3^+^ Treg cells among the CD4^+^CD25^+^ cells in SLE (**Figure 1C**). Collectively, although SLE CD4^+^ T cells are primed to receive and activate IL-2 signaling as evidenced by the upregulation of CD25 and increased IL-2-induced STAT5 phosphorylation under Treg-polarizing conditions, Treg cells are depleted in SLE patients (**Figure 1C**). However, it is corrected once cells are stimulated with anti-CD3/CD28 (**Figure 1A** (12, 30),) likely because of a large amount of IL-2 produced by T cells (30). These findings point to the IL-2 deficiency underlying the SLE Treg depletion (6).

### Interleukin-2 Elicits the Expansion of Interleukin-13-Producing CD8^+^ T Cells That Also Express Interleukin-5 and Interferon-γ

**Figure 2:**
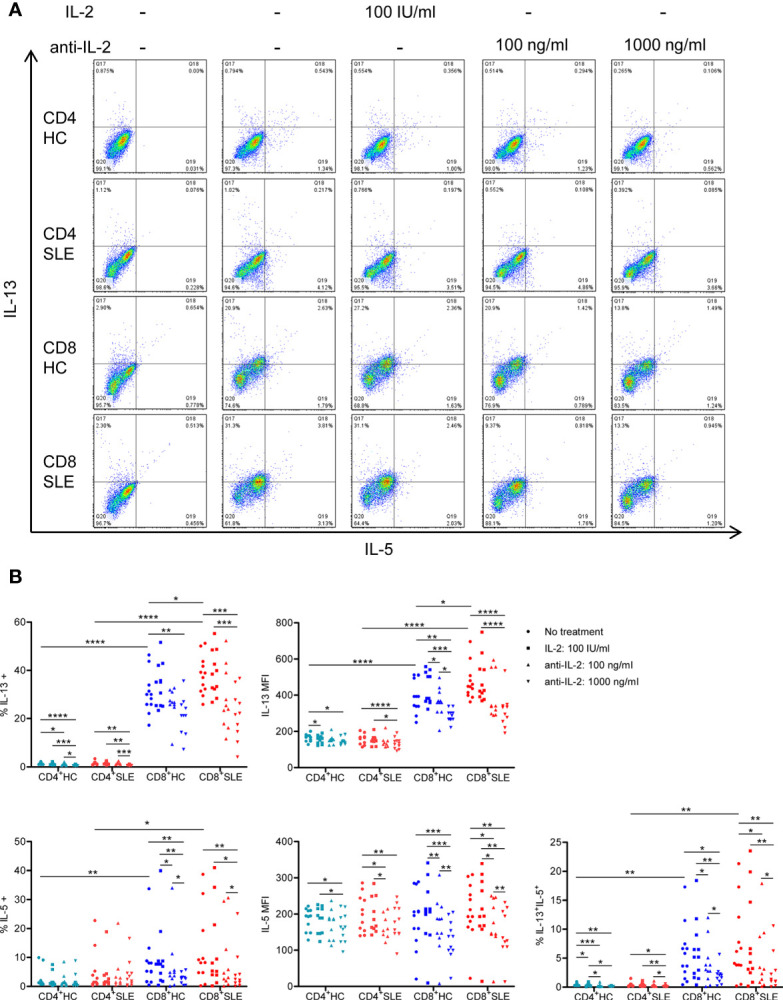
Systemic lupus erythematosus (SLE) CD8^+^ T cells produce increased IL-13 in an IL-2-dependent manner. **(A)** CD4^+^ and CD8^+^ T cells isolated from matched SLE and health control (HC) subjects were cultured for 3 days with anti-CD3/CD28 in the presence or absence of IL-2 (100 IU/ml) or anti-IL-2 (100 or 1,000 ng/ml). IL-5 and IL-13 expression was determined by flow cytometry. Representative flow cytometry dot plots are shown. The dot plots on the left end represent isotype control staining. **(B)** Cumulative data of MFI and the frequency of expression of individual cytokines from 12 pairs of matched SLE and HC subjects. Statistical analysis was made by two-way ANOVA followed by Bonferroni’s correction for multiple comparisons (*p<0.05, **p<0.01, ***p<0.001, ****p<0.0001).

**Figure 3:**
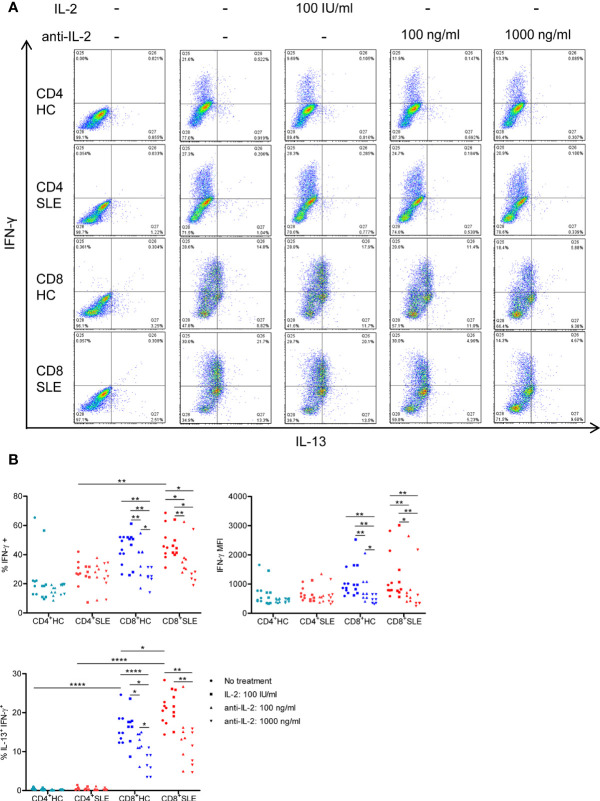
IL-2 expands IL-13^+^IFN-γ^+^ CD8^+^ T cells in systemic lupus erythematosus (SLE). **(A)** CD4^+^ and CD8^+^ T cells from matched SLE and health control (HC) subjects were cultured as described in **Figure 2**, and IL-13 and IFN-γ expression was determined by flow cytometry. **(B)** Cumulative data of mean fluorescence intensity (MFI) of IFN-γ and the frequency of IFN-γ^+^ and IL-13^+^IFN-γ^+^ cells. Statistical analysis was made by two-way ANOVA followed by Bonferroni’s correction for multiple comparisons (*p<0.05, **p<0.01, ****p<0.0001).

To evaluate the roles for IL-2 in pro-inflammatory cytokine expression in SLE T cells, magnetically isolated CD4^+^ and CD8^+^ T cells were cultured in the presence or absence of IL-2 or anti-IL-2. IL-2 positively controls the expression of IL-5, IL-13, and IFN-γ, but not IL-4, in particular, in CD8^+^ T cells (**Figures 2**, **3**, **S1**). CD4^+^ T cells appeared to be the predominant source of IL-21, and it was not IL-2 dependent (**Figure S2**). No meaningful IL-17 expression was observed (data not shown). Among these IL-2-dependennt cytokines, CD8^+^ T cells produced greater amount of IL-13 (HC: 30.41 ± 2.36%, 1.51 ± 0.14%, p<0.0001; SLE: 37.91 ± 2.27%, 1.53 ± 0.22%, p<0.0001) and IFN-γ (HC: 46.00 ± 3.22%, 25.07 ± 6.89%, p=ns; SLE: 48.04 ± 4.46%, 29.53 ± 2.83%, p<0.01) than CD4^+^ T cells (**Figures 2** and **3**). IL-13 expression in CD8^+^ T cells was greater than that in CD4^+^ T cells on a per cell basis as determined by the mean fluorescence intensity (MFI) (HC: 396.67 ± 26.54, 165.64 ± 6.10, p<0.0001; SLE: 470.42 ± 27.39, 158 ± 8.65, p<0.0001). Of note, SLE CD8^+^ T cells produced greater amount of IL-13 (% positive cells: SLE: 37.91 ± 2.27%, HC: 30.41 ± 2.36%, p<0.05; MFI: SLE: 470.42 ± 27.39, HC: 396.67 ± 26.54, p<0.05) than healthy control CD8^+^ T cells (**Figures 2** and **3A**).

We next turned to the correlation analysis of type 1 and 2 cytokine expression. IL-13 expression was greater in IL-5-expressing cells than IL-5-non-expressing cells both in CD4^+^ and CD8^+^ T cells (**Figure S3A**). Accordingly, the expression of IL-13 and IL-5 was positively correlated in CD8^+^ T cells (**Figure S3B**), yielding a IL-13^+^ IL-5^+^ CD8^+^ T cell population that contracted in association with IL-2 blockade (**Figure 2**). In contrast, the expression of IL-13 and IL-4 appeared to be mutually exclusive (**Figure S1A**), and there was no difference of IL-13 expression between IL-4-expressing and -non-expressing cells (**Figure S3A**). Unexpectedly, IL-13 expression in IFN-γ-expressing CD8^+^ T cells was greater than that in IFN-γ-non-expressing CD8^+^ T cells (**Figure S3A**). Along this line, there was a positive correlation between the expression of IL-13 and IFN-γ (**Figure S3B**), resulting in the expansion of IL-13^+^ IFN-γ^+^ double-positive CD8^+^ T cells in SLE (SLE: 20.53 ± 1.48%, HC: 16.15 ± 1.48%, p<0.05, **Figures 3A, B****)** which contracted in association with IL-2 blockade. Collectively, IL-2 expanded IL-13-expressing CD8^+^ T cells including an IL-13^+^ IFN-γ^+^ double-positive subset in SLE.

### Interleukin-13 and Interleukin-5 Expression Are Associated with Interleukin-2-Induced STAT6 Phosphorylation and GATA-3 Expression in CD8^+^ T Cells

**Figure 4:**
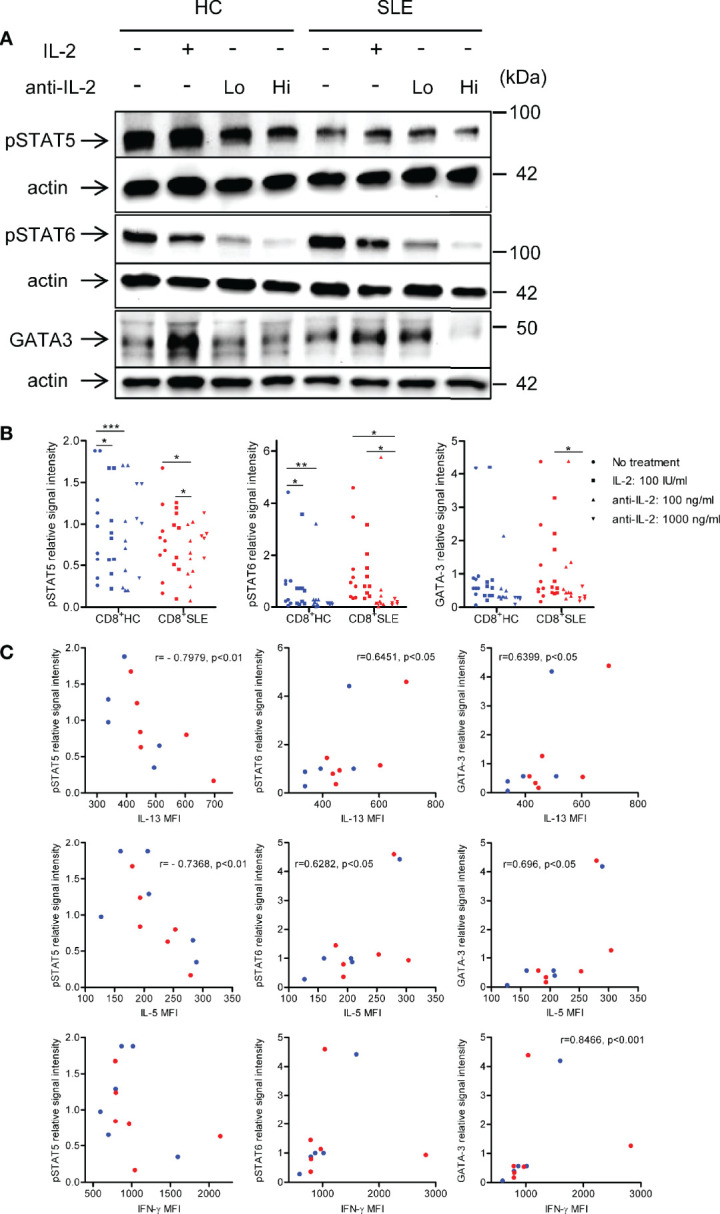
IL-2 induces STAT6 phosphorylation and GATA3 expression in systemic lupus erythematosus (SLE) CD8^+^ T cells. **(A)** CD8^+^ T cells from matched SLE and health control (HC) subjects were cultured as described in **Figure 2**. Expression of GATA-3 and phosphorylation of STAT5 at tyrosine 694 and STAT6 at tyrosine 641 were detected by immunoblotting. Representative immunoblot staining was presented. Lo and Hi concentrations of anti-IL-2 denote 100 and 1,000 ng/ml, respectively. **(B)** The signal intensity of phospho-STAT5, phospho-STAT6, and GATA-3 were normalized to that of actin. Cumulative data from 9 pairs of matched HC and SLE subjects. Data were analyzed by a two-tailed t-test (*p<0.05, **p<0.01, ***p<0.001). **(C)** Pearson’s and Spearman’s correlation analyses were performed to determine the association between the expression of cytokines (IL-13, IL-5, and IFN-γ) and transcription factors (phospho-STAT5, phospho-STAT6, and GATA-3). The blue and red plots represent data from HC and SLE patients, respectively. Spearman correlation coefficient was presented for the association between IL-13 and phospho-STAT5, IL-5 and phospho-STAT-5, and IFN-γ and GATA-3. Pearson correlation coefficient was presented for the remainder of associations.

To understand the mechanisms by which IL-2 induces the expansion of IL-13- and IL-5-producing CD8^+^ T cells, phosphorylation of STAT5 and STAT6 and GATA-3 expression were determined in the lysates of CD8^+^ T cells cultured in the presence or absence of IL-2 or anti-IL-2. Neutralization of IL-2 profoundly diminished the phosphorylation of STAT6 and GATA-3 expression in SLE CD8^+^ T cells; however, with regard to the impact of IL-2 blockade on STAT5 phosphorylation, such dose-dependent abolishment of phospho-STAT5 was not observed (**Figures 4A, B****)**. Of note, the expression of IL-13 and IL-5 were positively correlated with STAT6 phosphorylation and GATA-3 expression, but negatively correlated with STAT5 phosphorylation in CD8^+^ T cells (**Figure 4C**). In this respect, we previously documented increased GATA-3 expression in SLE CD8^+^ T cells as compared with healthy control CD8^+^ T cells (12). In contrast to these type 2 cytokines, IL-4 expression was not correlated with STAT5 or STAT6 phosphorylation or GATA-3 expression (data not shown). Unexpectedly, there was a positive correlation between the expression of IFN-γ and GATA-3 in CD8^+^ T cells (**Figure 4C**), which may account for the concurrent expression of IL-13 and IFN-γ (**Figure 3**). Our data collectively suggests that IL-2 induces IL-13, IL-5, and IFN-γ expression in lupus CD8^+^ T cells *via* STAT6-GATA-3 dependent mechanisms in contrast to the Treg differentiation in which IL-2-STAT5 axis plays a more essential role (**Figure 1**).

## Discussion

In this study, we documented that IL-2-STAT5 pathway was more critical to Treg differentiation in SLE than in health control subjects. On the other hand, IL-2 expanded IL-13-producing CD8^+^ T cells that also expressed IL-5 and IFN-γ in lupus patients *via* a signaling pathway likely involving the activation of STAT6-GATA-3 axis, but not STAT5. It is important to note that lupus CD4^+^ T cells were primed to receive and activate IL-2 signaling as evidenced by the upregulation of CD25 and enhanced IL-2-induced STAT5 phosphorylation during Treg differentiation even though the CD4^+^CD25^+^FOXP3^+^ Treg population was depleted in SLE patients. However, the Treg depletion is corrected by anti-CD3/CD28 stimulation *in vitro* (12, 30). A series of these findings pinpoint the IL-2 deficiency underlying the Treg depletion in SLE (6).

While it is not clear how IL-2 activates STAT6 and GATA-3 in CD8^+^ T cells, our data provides compelling evidence that this pathway is STAT5 independent. The neutralization of IL-2 did not have a robust impact on STAT5 phosphorylation unlike STAT6 phosphorylation and GATA-3 expression. In addition, the expression of IL-13 and IL-5 were negatively correlated with STAT5 phosphorylation in CD8^+^ T cells. It is worth noting here that IL-2-dependent STAT5 phosphorylation and GATA-3 expression are essential for IL-4-independent early IL-4 expression in CD4^+^ T cells (31). These observations suggest that IL-2 utilizes different signaling pathways in eliciting the activation and differentiation of CD4^+^ Treg cells, CD4^+^ non-Treg cells, and CD8^+^ T cells. Unlike the neutralization of IL-2, the supplementation of IL-2 had no effects on the expression of IL-5 and IL-13 and IFN-γ, which is attributed to a large amount of IL-2 produced by T cells in response to CD3/CD28 stimulation alone (30).

Although IL-2 activates the mechanistic target of rapamycin (mTOR), its blockade by sirolimus elicits Treg expansion both *in vitro* (30) and *in vivo* (32). This suggests that mTOR activation is not essential for IL-2-induced Treg expansion. IL-2 also activates the Akt-mTOR axis in CD8^+^ T cells (33). Our previous study showed that mTOR blockade restrained GATA-3 expression in FOXP3^-^CD4^+^ T cells (30). These findings suggest that the IL-2-Akt-mTOR axis may also affect GATA-3 expression in CD8^+^ T cells.

Among the three type 2 cytokines studied, there was a positive correlation between the expression of IL-13 and IL-5. IL-2 blockade abolished the expression of IL-13 and IL-5 in association with diminished STAT6 phosphorylation and GATA-3 expression. Additionally, the expression of these type 2 cytokines was positively correlated with STAT6 phosphorylation and GATA-3 expression. We previously reported increased GATA-3 expression in lupus CD8^+^ T cells (12). A series of these findings suggest that STAT6-GATA-3 axis drives the expression of IL-13 and IL-5 in CD8^+^ T cells. Conversely, the expression of IL-13 and IL-4 appeared mutually exclusive, and there was no appreciable size of IL-13^+^ IL-4^+^ population. Furthermore, IL-4 expression was resistant to IL-2-blockade-mediated abrogation of STAT6 phosphorylation and GATA-3 expression, and not correlated with these transcription factors. While our data does not exclude the involvement of STAT6-GATA-3 axis in the initial commitment to IL-4-expressing lineage as the study was not conducted on naïve CD8^+^ T cells, it suggests that CD8^+^ T cells maintain their IL-4 expression by STAT6-GATA-3-independent mechanisms.

While concurrent IFN-γ and IL-4 expression in CD4^+^ T cells has been reported (34), this is the first study documenting a dual expression of IL-13- and IFN-γ in CD8^+^ T cells. GATA-3 blocks IFN-γ expression (35, 36), whereas T-bet suppresses IL-4 and IL-5 expression in CD4^+^ T cells (37). Nonetheless, as such an antagonism has not been documented in CD8^+^ T cells, it is plausible that lupus CD8^+^ T cells employ a distinct molecular mechanism that allows for a dual IL-13- and IFN-γ expression. We identified a positive correlation between the expression of IFN-γ and GATA-3 in CD8^+^ T cells. Instead of proposing GATA-3 as a driver of IFN-γ expression in CD8^+^ T cells, we reason that IL-2 signaling activates a transcription factor upstream of both type 1 and type 2 programs. One such candidate is Notch as it was shown to concurrently regulate both Th1 and Th2 programs (38). Whether these cells in lupus patients are equipped with both helper- and cytotoxic functions would merit further investigation.

In conclusion, our data suggest that IL-2 supplementation may be a double-edged sword in the treatment of SLE, and supports the importance of Treg-cell-targeted delivery of IL-2. This study also challenged a long-held paradigm of type 1 and 2 cytokine antagonism by newly identifying IL-13^+^ IFN-γ^+^ CD8^+^ T cells that may potentially promote inflammation in SLE.

## Data Availability Statement

The original contributions presented in the study are included in the article/**Supplementary Material**. Further inquiries can be directed to the corresponding author.

## Ethics Statement

The studies involving human participants were reviewed and approved by SUNY UPSTATE IRB. The patients/participants provided their written informed consent to participate in this study.

## Author Contributions

Both authors participated in the design and analysis of the experiments and writing of the manuscript. HK executed the experiments. All authors contributed to the article and approved the submitted version.

## Funding

This work was supported by grants R01-AI072648 and AI122176 from the National Institutes of Health and the Department of Medicine at the SUNY Upstate Medical University.

## Conflict of Interest

The authors declare that the research was conducted in the absence of any commercial or financial relationships that could be construed as a potential conflict of interest.

## Supplementary Material

Supplementary Figure 1 IL-2 does not control IL-4 expression. **(A)** CD4^+^ and CD8^+^ T cells from matched SLE and HC subjects were cultured as described in **Figure 2** and IL-13 and IL-4 expression was determined by flow cytometry. **(B)** Cumulative data of MFI and frequency of IL-4^+^ cells. Statistical analysis was made by two-way ANOVA followed by Bonferroni’s correction for multiple comparisons (*p<0.05).

Supplementary Figure 2 CD4^+^ T cells produce greater amount of IL-21 than CD8^+^ T cells in an IL-2-independent manner. **(A)** CD4^+^ and CD8^+^ T cells from matched SLE and HC subjects were cultured as described in **Figure 2** and IL-13 and IL-21 expression was determined by flow cytometry. **(B)** Cumulative data of MFI and frequency of IL-21^+^ cells. Statistical analysis was made by two-way ANOVA followed by Bonferroni’s correction for multiple comparisons (*p<0.05, **p<0.01, ***p<0.001, ****p<0.0001).

Supplementary Figure 3 Expression of IL-13 positively correlates with that of IL-5 and IFN-γ. **(A)** CD4^+^ and CD8^+^ T cells from matched SLE and HC subjects were cultured as described in **Figure 2**. The MFI of IL-13 was compared between IL-5^+^ and IL-5^-^ cells, IFN-γ^+^ and IFN-γ^-^ cells, and IL-4^+^ and IL-4^-^ cells. Data were analyzed by a paired two-tailed t-test (*p<0.05, **p<0.01, ***p<0.001, ****p<0.0001). **(B)** Pearson’s and Spearman’s correlation analyses were performed to determine the association between IL-13 and IL-5 or IFN-γ. Pearson correlation coefficient was presented for the association of IL-13 with IL-5. Spearman correlation coefficient was presented for the association of IL-13 with IFN-γ.
